# Childcare Exposure to Severe Acute Respiratory Syndrome Coronavirus 2 for 4-Year-Old Presymptomatic Child, South Korea

**DOI:** 10.3201/eid2702.203189

**Published:** 2021-02

**Authors:** Yoonsun Yoon, Gwang-Jun Choi, Ji Yeong Kim, Kyung-Ran Kim, Hwanhee Park, Jae Kyung Chun, Yae-Jean Kim

**Affiliations:** Samsung Medical Center, Sungkyunkwan University School of Medicine, Seoul, South Korea (Y. Yoon, K.-R. Kim, H. Park, Y.-J. Kim);; Division of Infectious Disease Control, Daegu Metropolitan City, Daegu, South Korea (G.-J. Choi);; Miryang-si Public Health Center, Miryang, South Korea (J.Y. Kim, J.K. Chun)

**Keywords:** severe acute respiratory syndrome coronavirus 2, SARS-CoV-2, coronavirus, viruses, children, transmission, infection, childcare, exposure, presymptomatic, coronavirus disease, COVID-19, respiratory infections, zoonoses, South Korea

## Abstract

Data on transmission of severe acute respiratory syndrome coronavirus 2 (SARS-CoV-2) from preschool-age children to children and adults are limited. We investigated SARS-CoV-2 exposure at a childcare center in South Korea. A 4-year-old child, probably infected by his grandmother, attended the center during the presymptomatic period (February 19–21, 2020). Fever developed on February 22, and he was given a diagnosis SARS-CoV-2 infection on February 27. At the center, 190 persons (154 children and 36 adults) were identified as contacts; 44 (23.2%) were defined as close contacts (37 children and 7 adults). All 190 persons were negative for SARS-CoV-2 on days 8–9 after the last exposure. Two close contacts (1 child and 1 adult) showed development of symptoms on the last day of quarantine. However, subsequent test results were negative. This investigation adds indirect evidence of low potential infectivity in a childcare setting with exposure to a presymptomatic child.

The exact number of global confirmed cases of coronavirus disease (COVID-19) in children is unknown. Since the first case report in a child, 2.2%–6.7% of reported cases from China, the United States, the European Union, and the United Kingdom have occurred in children (*1**–**3*). In South Korea, 14,305 SARS-CoV-2 cases were diagnosed as of July 31, 2020 (*4*). Of these cases, 1,028 were in children <19 years of age; the proportions of cases for persons <19 years of age was 7.2% and for persons for <9 years of age was 1.7% (*4*). The proportion of COVID-19 cases in children in South Korea was higher than that for China (2.2%) and the United States (5.1%) (*1**,**2*). This proportion was comparable to that for the European Union and the United Kingdom (6.7%) (*3*).

Respiratory virus infection among young children in childcare settings is a major epidemiologic consideration. Children are believed to be vectors for transmission of many respiratory viral diseases, including influenza and infection with respiratory syncytial virus (*5**–**7*). However, there are few data on SARS-CoV-2 transmission among young children.

In South Korea, the first imported case of SARS-CoV-2 from Wuhan, China, was reported on January 20, 2020 (*8*). The first case in a child from South Korea was diagnosed on February 18 (*9*). A religious group–related large outbreak in Daegu (a city with a population of 2.4 million in the southeastern part of the country) started on February 18 (*9*); a total of 5,212 patients were eventually found to have COVID-19 related to the religious group outbreak (*10*). Social distancing measures, including temporary closure of childcare centers, were initiated in Daegu immediately. However, other regions with no confirmed patients with COVID-19 remained open, including Miryang-si (population 108,600, 60 km from Daegu). The first patient with COVID-19 in Miryang-si was a 35-year-old man given a diagnosis on February 26, when there were 710 confirmed cases in Daegu (*11*). Subsequently, his 4-year-old son was given a diagnosis of COVID-19. The child had attended a childcare center during his presymptomatic period (February 19–21). In this study, we report the results of an epidemiologic investigation of potential exposure to a presymptomatic child who attended a childcare center in South Korea.

## Materials and Methods

### Setting

On February 27, 2020, the Miryang-si Public Health Center was notified of a confirmed case of a 4-year-old child with COVID-19 and possible exposure of children and adult staff by this child at a childcare center. The childcare center consisted of 17 classes divided by age group from <1 to 7 years. Children usually stayed at the center from 8:00 am to 6:00 pm, depending on schedules of parents. The index case-patient attended a class for 4- or 5-year-old children that had 13 classmates and 2 teachers. Each classroom had its own toilet, and children ate lunch and snacks in their classrooms with classmates and teachers. The center also has shuttle buses that can transport an average of 20 children, 2 teachers, and 1 driver.

Under the guidance of local health authorities, wearing masks, more frequent hand hygiene, and disinfection of the environment were required before the child tested positive. Adult staff at the center wore masks, but mask wearing by children were not consistent.

### Epidemiologic Investigation and Case Definition

An epidemiologic investigation was conducted. Close contacts were identified by trained epidemic intelligence officers on the basis of surveillance by closed-circuit television, childcare schedules, and statements from teachers. Close contact was defined as a person who had face-to-face contact for >15 minutes or who had direct physical contact with the index case-patient. Persons who used the same shuttle bus were also considered to be close contacts.

### Response Measures

After the index case-patient was identified, the center was closed. All potentially exposed persons were quarantined at home for 14 days.

Symptoms of close contacts were actively monitored by the local health authority through phone calls twice a day. For the remaining persons, passive reporting through self-assessment for fever or a defined set of newly present symptoms indicative of COVID-19 was conducted. Acute respiratory symptoms included fever, sore throat, rhinorrhea, myalgia, dyspnea, or cough.

All children and staff members (n = 190) at the center were tested 8–9 days after the last exposure for SARS-CoV-2, which was the earliest time point on which we could perform PCR considering the median incubation period for COVID-19 (*12*). The tests were performed at a drive-through test facility (*13*) or the COVID-19 screening clinic of Miryang-si Public Health Center. All samples were collected by obtaining nasopharyngeal swab specimens and tested by using real-time reverse transcription PCRs for SARS-CoV-2.

### Data Collection and Analysis

We obtained information on demographic characteristics and presence of symptoms by using standardized epidemiologic investigation forms. The investigation was a part of a public health response and was not considered research subject to institutional review board approval; therefore, written informed consent was not required. Personal information was accessed only by the public health officer of Miryang-si and epidemic intelligence officer of Daegu. Participant confidentiality was maintained throughout the study.

## Results

### Family Exposure

The index case-patient was a 4-year-old boy who lived in Miryang-si and was suspected of contracting the virus from his grandmother, who lived in Daegu. His grandmother attended the religious services on February 12 and 16 that were related to the large religious group outbreak in Daegu. He had contact with his grandmother on February 12 and 15 when he visited Daegu with his father. However, he and his father did not attend the religious service. His grandmother also came to the child’s house and stayed in Miryang-si during February 17–27.

**Figure 1:**
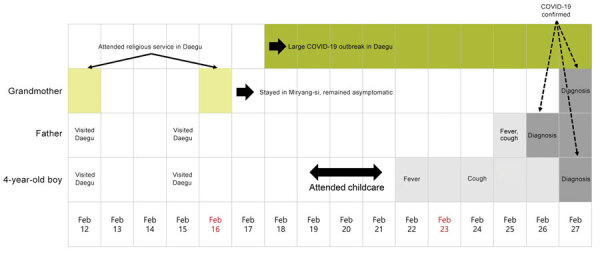
Timeline of family exposure and progress of symptoms until diagnosis of a 4-year-old child (index case-patient) and adult family members, South Korea. The index case-patient attended a childcare center during February 19–21, 2020. He showed development of fever on February 22. His father was given a diagnosis of COVID-19 on February 26. The index case-patient and his grandmother were given a diagnosis on February 27. The asymptomatic grandmother was suspected to be the primary case-patient in the family because she attended a religious service in Daegu, where a large outbreak occurred. Sundays are indicated in red. COVID-19, coronavirus disease.

The index case-patient showed development of fever (temperature 39°C) on February 22 and a cough on February 24. He was treated by a pediatrician on February 23 and 25. His father also showed development of fever, cough, and myalgia. The father was confirmed to have COVID-19 on February 26 (cycle threshold [C_t_] value for envelope gene 19.0, positive cutoff value 40.0). The child and his asymptomatic grandmother were confirmed to have COVID-19 on February 27. The C_t_ value was 24.6 (positive cutoff value 37.0) for the child and 32.7 (positive cutoff value 37.0) for the grandmother. His grandmother was asymptomatic during this entire period (Figure 1).

### Childcare Exposure

The index case-patient attended the childcare center on February 19–21 during a presymptomatic period before his fever developed (Figure 1). He traveled to the center by shuttle bus; the bus ride took »30 minutes in each direction. On the shuttle bus from his house to the center, 9 children, 2 teachers, and 1 bus driver were exposed. On the shuttle bus from the center to his house, an additional 15 children and 2 teachers were exposed. The boy attended the center for an average of »8 hours/day, and he had lunch and 2 snack times/day with his classmates in his classroom. He used the toilet in the classroom »5 times/day. There was no outside activity because of cold weather. Thirteen classmates of similar ages and 2 teachers were exposed to the index case-patient in the same classroom. Among these persons, 1 child was also exposed on the shuttle bus. Closed-circuit television review additionally identified a friend from another class who visited his classroom and played with him.

**Figure 2:**
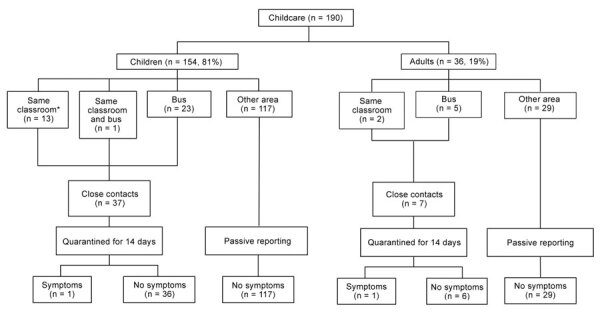
Persons exposed to severe acute respiratory syndrome coronavirus 2 (SARS-CoV-2) (154 children and 36 adults) in a childcare center and a shuttle bus, South Korea. All exposed persons underwent testing for SARS-CoV-2 and showed negative results. Close contacts (37 children and 7 adults) were quarantined for 14 days, and persons who had symptoms during the quarantine period were retested; all had negative results. *One child was exposed when she visited the classroom of the index case-patient.

A total of 190 persons (154 children and 36 adults) at the center were identified as potential contacts (Figure 2). The median age of exposed children was 4.1 years (range 0.9–7.2 years), and 75 (49%) were male. The median age of exposed adults was 42.0 years (range 22.1–64.8 years), and 3 (8%) were male. Of the 190 contacts, 44 (23.2%) were exposed to the index case-patient and considered close contacts: 37 (84.0%) children and 7 (16.0%) adults (1 bus driver and 6 teachers).

After the investigation, all 190 exposed persons had PCR testing for SARS-CoV-2 on the 8th and 9th days after the last exposure; 185 were tested in a drive-through test center, and 5 were tested in the COVID-19 screening clinic of Miryang-si Public Health Center. Among close contacts, 1 classmate and 1 teacher in the class of the index case-patient showed development of cough on the last day of quarantine (14 days from the last exposure). However, subsequent testing of nasopharyngeal swab specimens for these 2 persons showed negative results. The investigation and monitoring ended on March 6, 2020, which was 14 days after the last day the child attended the center, which was 1 day before fever onset. There were no laboratory-confirmed secondary cases of COVID-19 during this exposure. Although 2 close contacts showed development of symptoms during the quarantine period and were retested, test results for these close contacts were negative.

## Discussion

We describe a childcare center exposure of SARS-CoV-2 for a 4-year-old presymptomatic child and the subsequent investigation, with detailed information on exposure types and durations among exposed children and adult staff members. Among all 190 persons at the center who were tested and monitored, no secondary cases were identified.

There are few data on childcare exposure to SARS-CoV-2 among young children. Recently, a few reports were published from the United States and Australia. In Rhode Island, USA, 666 of 891 childcare programs reopened as of July 31, 2020 (*14*). Local health authorities required strict regulations, including restricting the maximum number of persons in a class, limiting switching between classes, staff use of masks, daily symptom monitoring of staff and attendees, and enhanced disinfection of the center (*14*). During June 1–July 31, a total of 52 laboratory-confirmed or probable COVID-19 cases, including cases in 30 children, were identified. Among the 666 reopened childcare centers, staff members and attendees from 29 (4.0%) centers were exposed to SARS-CoV-2 (*14*). Epidemiologic investigation showed that there was 1 case without further transmission at 20 (69.0%) of these 29 centers. In 5 (17.0%) of the 29 programs, 2–5 COVID-19 cases/program were identified; however, there was no evidence of childcare-related transmission. Childcare-related transmission occurred at 4 (14.0%) of the 29 programs (2–10 COVID-19 cases/program); 2 of these programs did not adhere strictly to regulations (*14*). Therefore, of the 86% of childcare programs in the study that had COVID-19 cases, there were no instances of secondary transmission. In addition, a 2-year-old child attended childcare for 6 days while potentially infectious and did not produce secondary transmission.

In Salt Lake City, Utah, USA, during April 1–July 10, 2020, small-to-large outbreaks occurred in 3 childcare facilities for which complete investigation data were available (*15*). All 3 outbreaks were linked to index cases in adult staff members (*15*). Childcare-related transmission occurred; there were 2–15 COVID-19 cases/facility (*15*). The facility that had 15 patients given a diagnosis of COVID-19 did not require wearing masks for staff members and children. A total of 12 children (age range 8 months–10 years) were probably infected with SARS-CoV-2 at childcare centers (*15*). A total of 83 household and nonhousehold contacts were exposed to these 12 case-patients. Among those 83 contacts, 5 probable and 7 confirmed patients with COVID-19 were identified, including parents and siblings (*15*).

In New South Wales, Australia, 10 childcare centers for children age 6 weeks–5 years had exposure to COVID-19 cases during January 25–April 10, 2020 (*16*). The primary cases were defined as initial infectious cases in this setting (*16*). Of those 10 centers, the exposure occurred by primary pediatric cases in 3 centers (30.0%). At these 3 centers, 85 children and 37 adults were defined as being close contacts and quarantined (*16*). Among these persons, 17 (20.0%) of 85 children and 11 (30.0%) of 37 adults were tested, and all showed negative results (*16*). Overall, secondary transmission occurred in only 1 center, in which the primary case-patient was a 49-year-old woman (staff member). Of 37 close contacts at that childcare center, 6 staff and 7 children were infected with SARS-CoV-2 (*16*). However, there was little evidence of child-to-child transmission or child-to-adult transmission in that epidemiologic investigation (*16*).

Our findings, along with literature discussed, might suggest potential low transmissibility of SARS-CoV-2 among young children in childcare settings. However, there might be differences in background COVID-19 situations between countries in which studies were conducted. Our epidemiologic investigation was conducted at the early stages of the pandemic, when a large outbreak first started in Daegu and the virus rapidly spread to nearby cities. Public awareness, mitigation measures, and public health responses were believed to be incomplete at that time. The study from Australia was conducted in communities that had low transmission rates and good public health responses (*17*). Although the studies from the United States showed that a few child-to-child or child-to-adult transmissions probably occurred in childcare centers, community transmission rates were higher in that country, which might confound true transmission rates from pediatric patients with COVID-19 in childcare settings. In addition, less strict adherence to precautions in some childcare facilities could have also affected the childcare-related transmission (*14*,*15*).

There were also a few reports of school exposure in older children during the early period of the COVID-19 pandemic. A report of a cluster in the French Alps included a 9-year-old boy who did not transmit SARS-CoV-2 to any other persons, although 112 persons at 3 different schools had contact with him (*18*). However, detailed information on exposure at schools was not available for that study. In a report from New South Wales, Australia, there were several high schools and 1 primary school in which many children and adolescents were exposed to a child (index-patient[s]) but few secondary cases resulted (*19*). In 15 schools (10 high school and 5 primary schools), 18 COVID-19 cases (in 9 students and 9 staff) were identified during March 5–April 3, 2020. Of 863 identified close contacts, only 2 secondary cases, both in children, resulted from transmission in the school setting; 1 case-patient was infected by another child and the other case-patient by an adult staff member. However, there were no details on exposure setting. In Ireland, for notifications of SARS-CoV-2 infection before school closure in March 2020, there was no transmission from 3 children (index case-patients) among 905 contacts in the school settings (*20*). All children who had COVID-19 in Ireland attended schools during the presymptomatic and symptomatic periods, and other children were exposed in a variety of settings, including music lessons (woodwind instruments) and choir practice, both of which are high-risk activities for virus transmission. Studies in France (*18*) and Ireland (*20*) performed laboratory tests only for symptomatic persons and might have underestimated asymptomatic or paucisymptomatic patients. In comparison, we collected test results on all persons at the childcare center in our study.

There have been reports demonstrating that children are not the main drivers of the COVID-19 outbreak (*21*,*22*), but it is unclear whether these findings are caused by low susceptibility, low transmissibility, or both. An age-structured mathematical model estimated that persons <20 years of age have lower susceptibility to SARS-CoV-2 infection than adults (*23*). Different immune responses or host factors of children have been also suggested as possible mechanisms of their low susceptibility (*24*,*25*). However, an epidemiologic study from China reported a conflicting finding that SARS-CoV-2 exposure rates for children were comparable to those for adults (*26*).

Because children who had COVID-19 had milder symptoms and a high proportion of subclinical infections, viral load and transmissibility during the asymptomatic or presymptomatic period is of particular interest (*23*,*27*,*28*). Several studies of adult patients showed that viral shedding of SARS-CoV-2 peaked at ≈1–2 days before symptom onset, and a substantial proportion of transmission probably occurred during presymptomatic or asymptomatic periods in the index case-patient (*29*,*30*). In children, SARS-CoV-2 RNA was also detected at a comparably high level, as in adults, at the time of diagnosis (*31*). However, more data are needed on whether young children have a high level of virus during the presymptomatic period, as in adults, and can transmit the virus to others.

In our study, the index child was present at a childcare for an average of 8 hours/day and had several meals/snacks with his young classmates at a close distance, with probable close physical contact. However, there were no additional cases.

This study had a few limitations. First, this study was a single epidemiologic investigation of SARS-CoV-2 exposure at 1 childcare center. More data on transmission from young pediatric index case-patients to other children and adults in educational settings are needed. Second, it was not proven that the index case-patient in this report was shedding virus during the presymptomatic period.

Closing childcare or schools has probably reduced transmission of SARS-CoV-2 in children (*32*). However, decisions regarding reopening schools or childcare centers are critical in many countries that are considering the social, educational, and economic benefits to society and children (*33*). Our investigation adds indirect evidence of low potential infectivity among children in a childcare setting when exposed to a presymptomatic child. Therefore, there might be a chance to safely reopen childcares if certain conditions are satisfied, including such infection prevention protocols as good personal hygiene practices, wearing masks, daily symptom monitoring of staff members and attendees, and disinfection of possibly contaminated surfaces and items.
